# Supplementary material for: Causal Relationship between Aging and Anorexia Nervosa: A White-Matter-Microstructure-Mediated Mendelian Randomization Analysis
Source: Biomedicines. 2024 Aug 16;12(8):1874. doi: 10.3390/biomedicines12081874 (PMC11351342; doi:10.3390/biomedicines12081874)
Supplement: Supplementary file 1 [file biomedicines-12-01874-s001.zip › Supplementary Figure/Figure S4/Singe SNP plot of MR analysis for TL to ubm-b-1670.pdf]

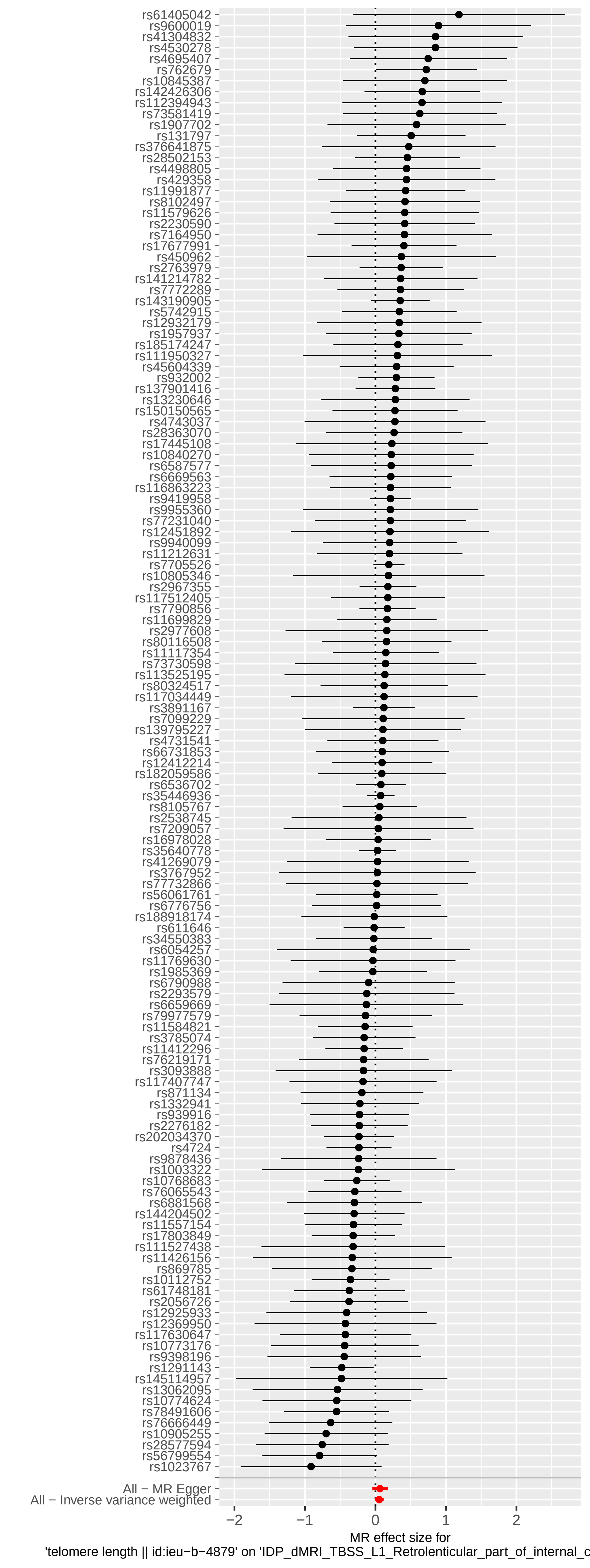
rs61405042
rs9600019
rs41304832
rs4530278
rs4695407
rs762679
rs10845387
rs142426306
rs112394943
rs73581419
rs1907702
rs131797
rs376641875
rs28502153
rs4498805
rs429358
rs11991877
rs8102497
rs11579626
rs2230590
rs7164950
rs17677991
rs450962
rs2763979
rs141214782
rs7772289
rs143190905
rs5742915
rs12932179
rs1957937
rs185174247
rs111950327
rs45604339
rs932002
rs137901416
rs13230646
rs150150565
rs4743037
rs28363070
rs17445108
rs10840270
rs6587577
rs6669563
rs116863223
rs9419958
rs9955360
rs77231040
rs12451892
rs9940099
rs11212631
rs7705526
rs10805346
rs2967355
rs117512405
rs7790856
rs11699829
rs2977608
rs80116508
rs11117354
rs73730598
rs113525195
rs80324517
rs117034449
rs3891167
rs7099229
rs139795227
rs4731541
rs66731853
rs12412214
rs182059586
rs6536702
rs35446936
rs8105767
rs2538745
rs7209057
rs16978028
rs35640778
rs41269079
rs3767952
rs77732866
rs56061761
rs6776756
rs188918174
rs611646
rs34550383
rs6054257
rs11769630
rs1985369
rs6790988
rs2293579
rs6659669
rs79977579
rs11584821
rs3785074
rs11412296
rs76219171
rs3093888
rs117407747
rs871134
rs1332941
rs939916
rs2276182
rs202034370
rs4724
rs9878436
rs1003322
rs10768683
rs76065543
rs6881568
rs144204502
rs11557154
rs17803849
rs111527438
rs11426156
rs869785
rs10112752
rs61748181
rs2056726
rs12925933
rs12369950
rs117630647
rs10773176
rs9398196
rs1291143
rs145114957
rs13062095
rs10774624
rs78491606
rs76666449
rs10905255
rs28577594
rs56799554
rs1023767
All − MR Egger
All − Inverse variance weighted
−2
−1
0
1
2
MR effect size for
'telomere length || id:ieu−b−4879' on 'IDP_dMRI_TBSS_L1_Retrolenticular_part_of_internal_c
